# Smarter through group living?

**DOI:** 10.3758/s13420-018-0335-0

**Published:** 2018-07-10

**Authors:** Tom V. Smulders

**Affiliations:** Centre for Behaviour and Evolution, Institute of Neuroscience, Newcastle University, Newcastle, UK

**Keywords:** Comparative cognition, Social intelligence hypothesis

## Abstract

Wild Australian magpies living (or growing up) in larger social groups take fewer trials to solve a battery of four cognitive tests than those living (or growing up) in smaller groups. The tests all draw on a common underlying factor, but is this factor cognitive or motivational?

## Introduction

Does living in larger social groups make you smarter? Note that the question is not whether the selective pressure of living in larger social groups selects for smarter individuals. Rather, the question is whether living in larger social groups actually ‘trains’ an individual to be smarter.

For Australian magpies (*Cracticus tibicen dorsalis*), Ashton, Ridley, Edwards, and Thornton (2018) claim the answer is ‘yes’! They tested 56 free-living, habituated magpies from 14 different social groups on a battery of four cognitive tests: a detour-reaching task (a test of inhibition), an associative-learning task (associate the colour of the lid with reward), a reversal-learning task (switching the colour-reward association from the previous task), and a spatial memory task (find the correct well in a ten-well board). They found that performance on the four tasks was related to each other, across individuals. In fact, a Principal Component Analysis identified a common factor explaining more than 60% of the variance in performance across tasks. The authors relate this factor to the concept of general intelligence (G) in humans. They then found that birds performed better (took fewer trials to solve the task) on all four tasks (and therefore also on the summary factor) if they lived in larger groups (the range was 3–12 animals per group). The authors concluded that animals living in larger groups were generally more intelligent/cognitively more advanced.

Whereas the outcome (differences in performance) cannot be argued with, the interpretation can (Rowe & Healy, 2014). A battery of four cognitive tests, administered to wild birds in the field, impressive as it is, is by no means comprehensive enough to jump to conclusions about general intelligence. For one thing, adding further tests (as was done in a similar study using North Island Robins (*Petroica longipes*); Shaw, Boogert, Clayton, & Burns, 2015) may well reduce the proportion of variance explained by the first factor. For another, all the tests were food-motivated. Therefore, birds living in larger groups might be more motivated by food, and therefore more focused when trying to solve the task. To give credit to the authors, they did look at several potential measures of food motivation, such as foraging efficiency, time to approach the task, and body mass. But all of these are also confounded by other factors (resp. food availability, neophobia and activity, to name a few). Unlike Shaw et al. (2015), Ashton et al. (2018) did not include a direct test of food motivation (e.g. how much they would eat from an *ad libitum* food source), which would have been very helpful to untangle this potential confound.

Food motivation may also explain the rest of the results from the paper. The authors went on to investigate how these group differences developed. They tested juvenile birds, hatched in the different groups, and followed them throughout their first year of life. Interestingly, while at 100 days of age (shortly after nutritional independence), the juveniles from the different groups did not differ from each other in task performance; by 200 and (even more so) by 300 days of age, the birds hatched in larger groups convincingly outperformed the birds from the smaller groups. Australian magpies stay in their natal group for up to a year, but then disperse to go and join non-territorial flocks (Higgins, Peter, & Cowling, 2006). Therefore, (adult) group size remained stable over the study period (these birds can live for several decades). This suggests that the number of adults are an important factor in this developmental pattern. What remains unclear is whether this is through the cognitive challenge of social interactions with so many adults, through the increased competition for food, or because birds form larger groups when resources are harder to come by. The latter two explanations could work through increased food motivation (although ecological challenges could increase cognitive abilities as well). Indeed, such food motivation would increase as the juveniles get older (and are not supplemented by their parents anymore), in line with the observed developmental effect. A really interesting (but practically very difficult) follow-up would be to test these dispersed juveniles in their non-territorial flocks, where presumably birds from small groups and those from large groups join together. Is this group difference a permanent trait (as would be expected from the development of cognitive systems), or is it a response to current conditions (as might be expected if the difference is motivation-based)? And did the current adults living in large groups themselves grow up in large groups, or is there no connection between group size during development and breeding group size in adulthood?

Finally, Ashton et al. (2018) also address natural selection for cognitive traits. They find that females who perform better on the cognitive tests are more likely to hatch young, fledge young and raise fledglings to independence. Group size does not seem to cause this relationship, despite the fact that females from larger groups do better on the cognitive tests. The authors interpret this as evidence that cognitive differences can have fitness consequences, and therefore be selected for. They are careful not to claim that there is natural selection for cognitive traits going on in this particular case, since they also concluded that the cognitive differences in this study are driven by the developmental environment. However, food motivation (of the mother) could explain the fitness effects as well, if females with higher food motivation also obtain more food. Provisioning rate did not correlate significantly with cognitive performance, but the sample size was small (they only had provisioning rates for 11 females), and the correlation coefficient was large and positive (r=0.534), with a p-value just missing significance (p=0.09). We can therefore not rule out that the fitness benefit of higher-performing females is due to higher provisioning rates. Whether provisioning rates are higher because of higher food motivation or because of better cognitive skills remains open for debate.

In conclusion, Ashton and colleagues carried out a heroic task, testing cognition of wild, free-living birds and relating it to life-history variables. However, the study also highlights the importance of controlling for purely motivational factors in cognitive testing (Rowe & Healy, 2014). Like any observational study, there remain alternative explanations and future questions to be answered, but the methodology (and indeed the particular study population) provides ample opportunities to pursue these further questions in the future.
